# Significance of Human Papillomavirus in Head and Neck Cancers

**DOI:** 10.6004/jadpro.2015.6.3.7

**Published:** 2015-05-01

**Authors:** Kristy Lynn Boggs

**Affiliations:** University of South Florida, Tampa, Florida

Over 45,000 Americans are estimated to be newly diagnosed with cancer of the oral cavity and pharynx in 2015, and an estimated 8,650 will die of the disease (Siegel, Miller, & Jemal, 2015). It is well known that the combination of smoking and alcohol use increases the risk for developing head and neck squamous cell carcinoma (HNSCC). Head and neck squamous cell carcinoma is a general term that encompasses cancers of the nasal cavity, sinuses, lips, mouth, salivary glands, throat, and larynx. HNSCC has been considered to be a cancer predominantly seen in men of older age (National Cancer Institute [NCI], 2013).

The current trends, deaths, and survival rates demonstrate a modest decrease in HNSCC from 1975 to 2006 (NCI, 2015). However, despite this general decrease, research demonstrates that oropharyngeal-specific cancers are rising (American Cancer Society, 2015; Gillison et al., 2012).

Currently, there is evidence that tissue collected from patients diagnosed with head and neck cancer may be positive for human papillomavirus (HPV) DNA, particularly cancers of the oropharyngeal site (Mehanna et al., 2013). The oropharynx refers to the middle part of the throat behind the mouth, including the back third of the tongue, the soft palate, the side and back walls of the throat, and the tonsils (NCI, 2015).

According to the Centers for Disease Control and Prevention (CDC, 2013), 79 million people have HPV, and over 14 million cases are newly diagnosed each year. This virus is associated with cancer of the cervix, vagina, penis, and anus. The exact incidence of HPV-positive HNSCC is unknown and could be largely misrepresented because current practice does not support testing all HNSCC for HPV status.

Currently, the Surveillance, Epidemiology, and End Results Program (SEER) does not specify HPV status in statistics on oral cavity and pharynx cancers. However, according to the CDC (2014a), an estimated 72% of oropharyngeal cancers may be attributed to HPV infections, with more than 2,370 new cases diagnosed in women and nearly 9,356 new cases diagnosed in men every year in the United States.

## PATHOPHYSIOLOGY

Over 100 different subtypes of HPV have been discovered (CDC, 2012). Most subtypes cause common skin warts. These types are categorized as low or high risk. Low-risk types, such as HPV-6 and HPV-11, can cause benign papillomas (commonly called warts). Generally, infections with these types tend to resolve themselves spontaneously within 12 months.

However, high-risk types such as HPV-16 and HPV-18 can lead to chronic infection, cellular abnormalities, and then cancer. HPV-16 is linked to HNSCC of the oropharynx (American Cancer Society, 2015). Approximately 90% of HPV-related oropharyngeal cancers are positive for the HPV-16 subtype (Blitzer, Smith, Harris, & Kimple, 2014; D’Souza, Cullen, Bowie, Thorpe, & Fakhry, 2014; Gan, Zhang, Guo, & Fan, 2014; Gillison et al., 2012). Commons symptoms may vary and can be vague (NCI, 2015; see Table).

**Table 1:**
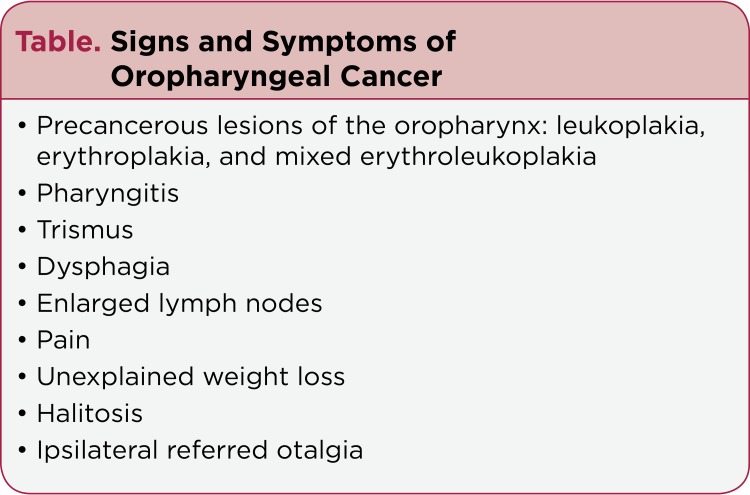
Signs and Symptoms of Oropharyngeal Cancer

**Figure 1:**
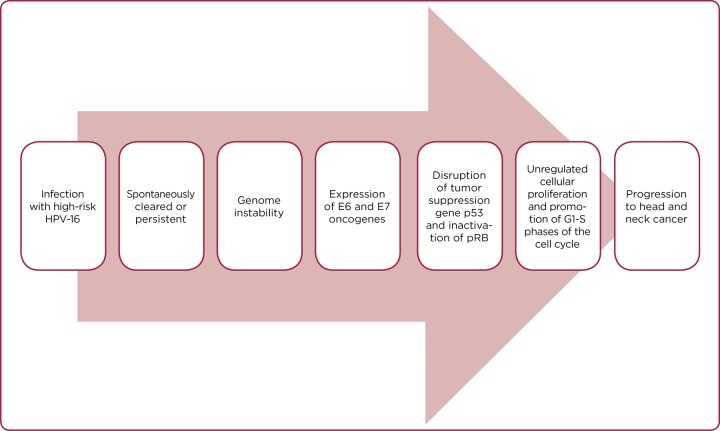
Mechanism of HPV-16 and tumorigenesis. HPV-16 = human papillomavirus subtype 1; pRB = protein retinoblastoma.

Infection with high-risk types of HPV can cause cells to make two proteins: E6 and E7. These proteins can turn off certain genes that control cell proliferation. Uncontrolled cell proliferation or cell growth can lead to cancer (American Cancer Society, 2015; Blitzer et al., 2014; see Figure 1).

## RISK FACTORS

**Sexual Behavior**

Human papillomavirus infection can spread from one person to another through sexual contact, including vaginal, anal, and oral sex. Oral sexual behavior and open-mouth kissing have been linked to an increased risk for oropharyngeal infection ( D’Souza., 2014; Geiger et al., 2014). In addition, similar to cervical cancer in women, the number of sexual partners can increase the risk for an HPV-positive tumor of the head and neck (American Cancer Society, 2015; CDC, 2013;  D’Souza., 2014; Geiger et al., 2014; Gillison et al., 2012; Kero et al., 2014a).

Current data indicate that oropharyngeal cancer is more prevalent in men than in women (CDC, 2013). D’Souza et al. (2014) found that sexual behavior alone accounted for these differences, with men tending to have more lifetime sexual partners than women. However, Gillison et al. (2012) demonstrated that after adjusting for high-risk sexual behavior, men continued to be at higher risk than women. To date, no strong evidence exists to explain why men are more affected by HPV-positive oropharyngeal cancers than women (Giuliano et al., 2014). The variation in prevalence surrounding gender and anatomic sites affected deserves further investigation.

**Smoking**

Smoking is a known carcinogenic that can cause changes in the DNA of cells and contribute to the increased risk for many types of cancers. Several studies demonstrate that smoking status can contribute to the persistence of HPV infection (Daling et al., 2004; Haukioja, Asunta, Söderling, & Syrjänen, 2014; Kero et al., 2014b). Smoking is considered an independent risk factor for HPV infection (Gillison et al., 2012).

Precisely how smoking contributes to chronic HPV infection and its progression to malignancy remains elusive. Castle (2008) discussed some plausible theories. First, smoking decreases immunity, which can reduce the body’s ability to effectively clear HPV infection. This, in turn, causes chronic infection and inflammation. Second, infection with high-risk HPV subtypes can cause uncontrolled cellular proliferation. These molecular changes combined with the carcinogens in tobacco smoke can work synergistically to encourage cancer development.

In addition, studies have indicated that smoking can adversely affect response to treatment and prognosis (Haughey & Sinha, 2012; Jensen, Jensen, & Grau, 2007; Moreira et al., 2014; Olthof et al., 2015). A review of the literature by Sobus and Warren (2014) revealed that smoking encourages further proliferation, metastasis, angiogenesis, and reduced apoptosis in cancer cells. This process can cause poor treatment response, increased recurrence of primary tumors, and increased mortality.

**Oral Hygiene**

There may be an association with poor oral hygiene that leads to an increased risk of developing HPV-positive HNSCC (Bui, Markham, Ross, & Mullen, 2013; Tezal et al., 2012). Poor oral hygiene leads to the accumulation of bacteria and microorganisms in the mouth, causing chronic inflammation, mucosal damage, and some microulceration.

Chronic inflammation plays several key roles in the development of cancer. According to Bonomi et al. (2014), chronic inflammation causes specific substances normally released during inflammation to facilitate healing to be constantly activated. These substances can then aid in tumor progression through increased proliferation, angiogenesis, and carcinogenesis. In addition, inflammation causes hypoxia to tissues resulting in DNA damage, leading to gene mutations that promote tumorigenesis.

## MANAGEMENT

In 2014, the National Comprehensive Cancer Network (NCCN) recommendations included testing tumors for HPV in the initial workup of oropharyngeal cancers (NCCN, 2014). Recommendations include either an immunohistochemistry for analysis of p16 expression or HPV in situ hybridization for detection of HPV DNA in tumor cell nuclei (NCCN, 2014). Testing is not considered in management decisions, however, and is used for prognosis purposes.

Current treatment guidelines, depending on stage, consist mainly of surgery, radiation therapy, chemoradiation therapy, and targeted therapies (National Cancer Institute, 2015). In a majority of studies, HPV-positive patients showed better prognosis and 5-year survival than HPV-negative patients with HNSCC (Ang & Sturgis, 2012; Chaturvedi, Engels, Anderson, & Gillison, 2008; Dufour, Beby-Defaux, Agius, & Lacau St. Guily, 2012; Fakhry et al., 2008; Ljokjel et al., 2014; Mirghani et al., 2014). It is speculated that the current guidelines for treatment could be minimized, given the more favorable prognosis of HPV-positive cancers, which subsequently could minimize side effects without affecting prognosis (Geiger et al., 2014; Masterson et al., 2014). However, there is currently insufficient evidence to change treatment guidelines based on HPV status in HNSCC (Masterson et al., 2014; Mehanna, Olaleye, & Licitra, 2012).

**Vaccines**

There are currently two HPV vaccines approved by the US Food and Drug Administration (FDA): Gardasil and Cervarix. Gardasil is quadrivalent and protects against HPV subtypes 6, 11, 16, and 18. Subtypes 6 and 11 are associated with genital warts. Cervarix is bivalent and protects against oncogenic types 16 and 18. These vaccines are currently approved to prevent cervical cancer and precancers in women.

The FDA has approved both vaccines to be given to girls between the ages of 9 and 26 and has currently approved Gardasil for boys between the ages of 9 and 21 to prevent genital warts. For women older than 26 and men older than 21 who did not receive the vaccine or did not receive all three doses, the vaccine can still be given. The CDC currently recommends that girls receive three doses of either vaccine at the age of 11 or 12. The future impact these vaccines may have on the prevalence of HPV-positive tumors of the head and neck is thus far undetermined.

**Psychosocial Impact**

Human papillomavirus has only newly been associated with cancers of the head and neck. The impact of discussing HPV results with patients and their partners has not been well studied (Baxi et al., 2013). Current research or information regarding strategies for discussion is minimal, thus there are currently no established guidelines for discussing HPV as it relates to oropharyngeal cancer. However, numerous studies have demonstrated that many women diagnosed with high-risk HPV-positive cervical cancers have experienced considerable psychological stress related to their diagnosis. Similar distress was discovered in men being tested and diagnosed with HPV-positive HNSCC (Cook, 2014; Daley et al., 2012; Harvey-Knowles & Kosenko, 2012).

Patients newly diagnosed with HPV-positive HNSCC can experience misconceptions and uncertainty regarding its transmission, latency, and future spread (Baxi et al., 2013). In a report examining the cognitive and emotional responses associated with HPV test results, the men who tested positive experienced negative emotional responses and were less likely to disclose the diagnosis with current or future partners (Daley et al., 2012). Furthermore, an exploratory study revealed that most patients lacked the knowledge connecting HPV status to their current cancer diagnosis and expressed interest in additional education (Milbury, Rosenthal, El-Naggar, & Badr, 2013). A systematic review regarding appropriate counseling messages for patients with HPV and associated conditions provided suggestions for clinical providers to lead such a discussion (Dunne, Friedman, Datta, Markowitz, & Workowski, 2011).

There are some commonalities between these suggestions and the CDC’s current key messages for women receiving cervical cancer screening and HPV testing (2011). For instance, it is important to deliver information in both verbal and written formats within a neutral, nonstigmatizing context. Teaching should focus on prevalence, signs and symptoms, and risk to partners. To reduce possible accusations between couples, providers must emphasize that HPV can remain dormant for many years and that having HPV does not imply infidelity, nor should it necessarily raise concerns about a partner’s health (CDC, 2011). It is reasonable to consider that similar topics will be useful in patients newly diagnosed with HPV-positive HNSCC. All clinical providers should aim to decrease any feelings of stigmatism or guilt that patients frequently experience with the diagnosis of a sexually transmitted infection (STI).

**Figure 2:**
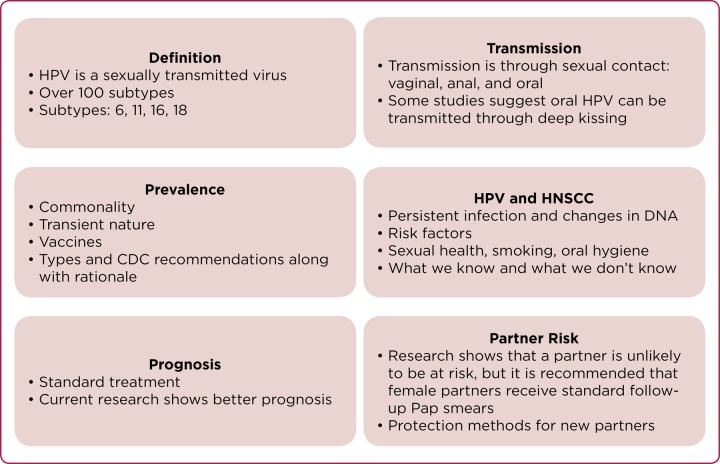
Suggested discussion points for having conversations with patients about the human papillomavirus. HPV = human papillomavirus; CDC = Centers for Disease Control and Prevention; HNSCC = head and neck squamous cell carcinoma

Moreover, what makes this even more challenging for these patients is being told that not only do they have cancer but that it may be related to an STI they contracted. This can add additional distress and confusion. Some possible strategies and key messages to convey to patients might include the prevalence of HPV, its transmission and prognosis, partner risk, and limitations in our present knowledge (Evans & Powell, 2014; Fakhry et al., 2008; Geiger et al., 2014; Kosenko, Craig, & Harvey-Knowles, 2012; see Figure 2).

## CONSIDERATIONS FOR THE ADVANCED PRACTITIONER

Currently, there are apparent gaps in knowledge regarding HPV-positive HNSCC. As research continues to expand, our understanding of the impact of these types of cancers will continue to change and develop. Discussion regarding the significance of an HPV-positive cancer can be complex and confusing. Future studies can help to guide advanced practitioners (APs) to identify key messages pertinent to patients and the most effective approaches to successful communication. It is imperative that the AP understand the impact of HPV on cancer development in all areas of medicine. Understanding the impact of HPV-related cancers and associated risk factors should guide the physical exam and informative discussions during patient visits. Identifying risk factors can aid in prevention and early detection in the general public. Educating the public and patients regarding risk factors for HPV can help reduce infection rates and thus prevent cancer from developing.

The oncology AP, in particular, may be the initial provider to inform patients about HPV testing, diagnosis, and prognosis as it relates to their cancer. It is important that these providers appreciate the common challenges and concerns patients may experience to enhance effective communication, reduce misconceptions, and ensure compliance with treatment.

Thus, it is essential for the AP to keep up with the developments in HPV diagnosis as they relate to specific cancers. The AP is in the unique position of having the ability to care for and foster trusting relationships with their patients and their families, as well as possessing advanced knowledge in medical practice. Consequently, the AP is the most appropriate provider to lead the way for future research exploring the psychological impact in the diagnosis of HPV-positive HNSCC.
